# X-ray photoemission analysis of clean and carbon monoxide-chemisorbed platinum(111) stepped surfaces using a curved crystal

**DOI:** 10.1038/ncomms9903

**Published:** 2015-11-12

**Authors:** Andrew L. Walter, Frederik Schiller, Martina Corso, Lindsay R. Merte, Florian Bertram, Jorge Lobo-Checa, Mikhail Shipilin, Johan Gustafson, Edvin Lundgren, Anto´n X. Brión-Ríos, Pepa Cabrera-Sanfelix, Daniel Sánchez-Portal, J. Enrique Ortega

**Affiliations:** 1 Donostia International Physics Centre, Paseo Manuel de Lardizabal 4, 20018 San Sebastian, Spain; 2 Brookhaven National Laboratory, Photon Sciences Directorate, NSLS II, Upton, New York, New York 11973, USA; 3 Centro de Física de Materiales CSIC/UPV-EHU-Materials Physics Center, Manuel Lardizabal 5, 20018 San Sebastian, Spain; 4 IKERBASQUE, Basque Foundation for Science, E-48011 Bilbao, Spain; 5 Department of Physics, 22 100 Lund University, Lund, Sweden; 6 Departamento Física de Materiales, Universidad del País Vasco, Apdo. 1072, 20080 San Sebastián, Spain; 7 Departamento Física Aplicada I, Universidad del País Vasco, 20018 San Sebastian, Spain

## Abstract

Surface chemistry and catalysis studies could significantly gain from the systematic variation of surface active sites, tested under the very same conditions. Curved crystals are excellent platforms to perform such systematics, which may in turn allow to better resolve fundamental properties and reveal new phenomena. This is demonstrated here for the carbon monoxide/platinum system. We curve a platinum crystal around the high-symmetry (111) direction and carry out photoemission scans on top. This renders the spatial core-level imaging of carbon monoxide adsorbed on a ‘tunable' vicinal surface, allowing a straightforward visualization of the rich chemisorption phenomenology at steps and terraces. Through such photoemission images we probe a characteristic elastic strain variation at stepped surfaces, and unveil subtle stress-release effects on clean and covered vicinal surfaces. These results offer the prospect of applying the curved surface approach to rationally investigate the chemical activity of surfaces under real pressure conditions.

## Abstract (web summary)

Systematic variation of surface sites may allow for more efficient testing of surface chemical reactions. Here, the authors use a platinum curved crystal and, by carrying out photoemission scans, are able to systematically address the fundamental CO-chemisorption process on a ‘tunable' vicinal surface.

## Graphical abstract

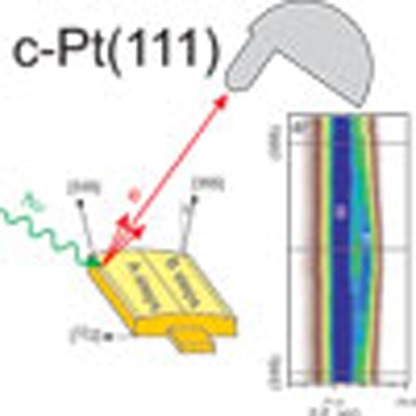

Many technologically relevant surface phenomena, such as crystal growth and chemical reactions, are known to be influenced by atomic steps. This is explained by the lower coordination of step atoms, which are preferred adsorption places, and hence nucleation centres, for example, for metastable phases that catalyze important chemical processes^1^,^2^. In reality, gas-surface interactions have complex, space-time oscillatory kinetics^3^, making it not *a priori* clear whether steps will promote or disturb a given reaction. Vicinal surfaces, that is, crystal surfaces close to a high-symmetry orientation, are frequently used for such chemistry studies, since they feature dense arrays of steps. Using crystals with curved shape one could selectively test different vicinal surface planes on the same sample, allowing a rational assessment of the role of steps on surface properties. Yet curved crystals (mostly, cylindrical) have been scarcely used to investigate critical physical-chemistry problems that involve steps (see ref. 4 for the carbon monoxide (CO)/Pt case, and new experiments in refs 5, 6, 7). The reason is that curved crystals, particularly full-cylinders or spherical samples^4^,^5^,^6^, add restrictions to *in situ* processing and to the use of surface-sensitive techniques. However, technical limitations can be significantly overcome with a reduced cylindrical section around a high-symmetry direction^7^,^8^,^9^,^10^. This allows a thorough analysis of vicinal planes making use, and hence benefiting from the most sophisticated and accurate surface science probes, such as high-resolution X-ray photoemission spectroscopy (XPS).

The power of our refined curved surface approach is demonstrated here for the model CO/Pt(111) system. A thorough statistical scanning tunnelling microcopy (STM) analysis straightforwardly probes the universal transition from entropic to elastic step interactions occurring at vicinal surfaces. By scanning the photon beam in XPS experiments we image, across the curved surface, the Pt 4*f* and C 1*s* core-levels at the clean and the CO-covered sample. In the clean curved surface, XPS scans allow measuring a subtle core-level energy shift, revealing that the tensile stress of the (111) plane is released in the presence of steps. For the CO-chemisorbed system we probe, with unprecedented resolution, the hierarchy of CO-chemisorption sites at different crystal planes, and also unveil a characteristic C 1*s* shift, likely due to a step-induced compressive-stress-release of the CO-saturated (111) surface.

## Results

### Sample preparation

**Figure 1:**
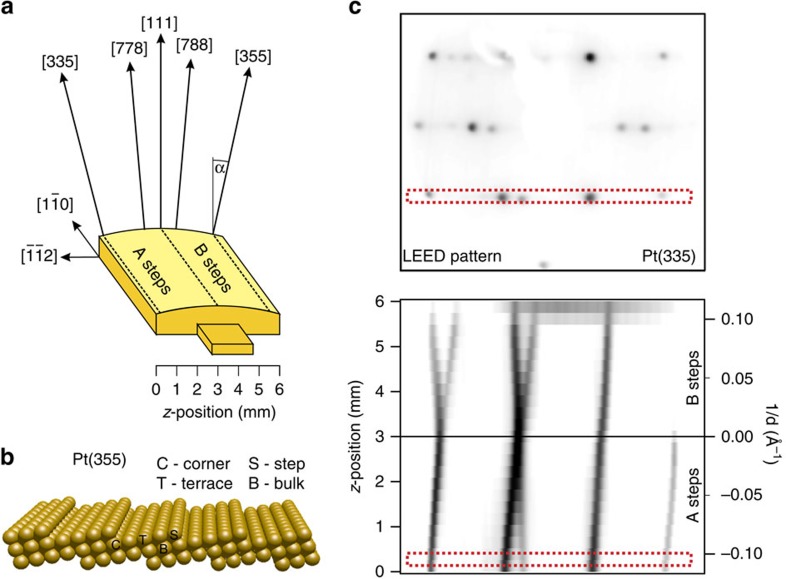
Geometry and low energy electron diffraction (LEED) scan of the curved Pt(111) surface. (**a**) Drawing of the Pt curved crystal used in the present investigation. It allows a smooth transition through the indicated miscut surfaces by moving the crystal along the bottom *z*-scale. (**b**) Sketch of the (355) plane, with different atomic coordinations *n* indicated, that is, bulk (B, *n*=12), corner (*C*, *n*=11), terrace (T, *n*=9) and step (*S*, *n*=7). (**c**) Top, representative LEED pattern taken from the (355) plane of the curved Pt crystal, and bottom, image ‘collage' showing the evolution across the curved crystal of the LEED intensity profile along (11)-(01)-(-11), as marked by the rectangle on the top pattern. The step density scale on the right side is deduced, at each position, from the size of the spot splitting relative to the distance between main diffracted beams^9^.

The sample is a cylindrical section cut and polished around the (111) plane (Fig. 1a). The total ‘miscut' angle range *α*=±15° allows examining a full family of A-type and B-type stepped surfaces vicinal to the [111] direction at the left and the right sides of the sample, respectively. One can smoothly switch from, for example, the (335) vicinal surface at *α*=+14.4°, which exhibits *d*=9.1 Å wide terraces (3+2/3 atomic rows) and {100}-like steps (A-type), to the (355) plane at *α*=−12.3°, *d*=10.6-Å wide terraces (4+1/3 atomic rows) and {111}-like steps (B-type). A reduced size (6 × 6 mm^2^) and adequate curvature of the sample is essential to facilitate mounting and processing in vacuum, while providing the accessibility to the distinct crystal directions with STM. More importantly, the sample design is key to enable a tunable X-ray beam of a synchrotron, to be used as a ‘local' scanning probe for high-resolution XPS. In this case, a standard 100-micron light spot size defines a maximum angular spread of the beam of Δ*α*=0.25° arc on the curved surface^11^, which allows one to carry out accurate terrace width *d*-dependent core-level analysis.

### LEED imaging of the clean surface

The structure of the curved surface is initially explored with low energy electron diffraction (LEED). In LEED experiments, we move the sample position laterally (‘*z*' scale in Fig. 1a) to directly probe the different surface planes with a 0.3 mm broad, 60-eV electron beam. The LEED pattern in Fig. 1c corresponds to the (335) surface, and exhibits the characteristic splitting of the diffracted beams observed in stepped surfaces. The dotted, red rectangle indicates a selected line over this LEED pattern, crossing the (11), (01) and (-11) spots. The ‘collage' image below is then built with individual (11)-(01)-(-11) line profiles acquired in Δ*z*=0.5 mm-steps across the curved surface. The resulting *z*-scan image reflects the linear variation of the spot splitting at both sides of the crystal^7^,^8^,^9^. Thus, the Pt(111) curved surface is microscopically defined by monatomic step arrays with smoothly varying step density and negligible step bunching or faceting, as sketched for the (335) surface in Fig. 1b. On the other hand, the size of the splitting in the LEED profile image of Fig. 1c straightforwardly defines the smooth step density, 1/*d* scale, which we will use in the XPS scan.

### STM imaging and analysis of the clean surface

**Figure 2:**
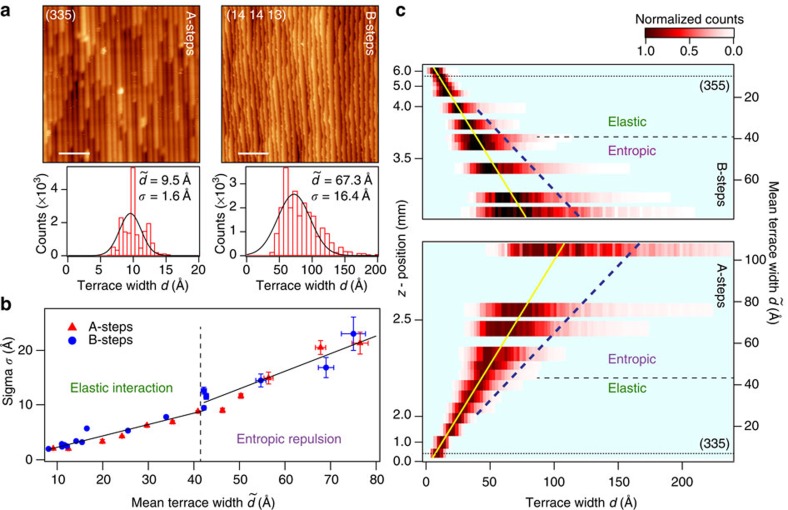
Scanning tunnelling microscopy (STM) analysis of the curved Pt(111) surface. (**a**) STM images at selected surface orientations, showing step lattice variations at the atomic scale. The scale bar in the (335) image is 6 nm, while for the (14 14 13) is 60 nm. The statistical analysis of the terrace width leads to the bottom histograms, both fitted with gaussian lines. This determines the mean terrace width 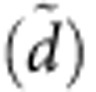 and the standard variation (σ) in each case. (**b**) *σ* versus 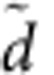 plot with data points from the analysis of full set of STM images taken across the curved surface. Error bars refer to the numerical precision determined by the fitting programme. A straight line fits the data (slope *m*) up to a critical value (dotted vertical line), suggesting a transition between elastic and entropic step interaction regimes. (**c**) Image plot of the curved surface built with all the probability histograms at different mean terrace 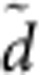 values. Histograms are individually normalized to the maximum probability. The dotted line marks the width of the gaussian distribution, from which the probability distribution departs in the entropic regime (see the text).

The structure of our tunable Pt(111) step array is analyzed with nanoscopic precision using STM. Of particular interest is the equilibrium shape of the steps and their distribution. As reflected in the images of Fig. 2a, the high mobility of step atoms at 300 K makes step edges look frizzy, leading to a local terrace width *d* variation around the average 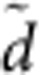 value. The statistical probability of *d* within each of the images is analyzed through the corresponding histograms, on top of which gaussian fits are shown. An ample set of STM images and histograms taken across the curved crystal are displayed in Supplementary Figs 1 and 2. Images in Fig. 2a are selected to illustrate two types of step distributions, which respectively characterize high and low step densities. In the low step density image (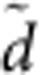
=67.3 Å), one can clearly observe the step meandering caused by thermal excitation of kinks. Assuming the condition that two steps cannot cross each other, the proximity of two steps reduces the allowed number of configurations, leading to an effective entropic repulsion^12^, and hence to an asymmetric probability distribution^13^. At the high step density 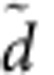
=9.5 Å steps look much straighter. This occurs because stronger 1/*d*^2^ step–step elastic interactions set in, making the probability distribution sharper and symmetric, that is, gaussian-like^12^.

Independently of the nature of the stepped crystal, the probability distribution of steps is expected to evolve from sparse arrays to dense lattices, as elastic interactions take over the entropic repulsion^14^. The curved surface allows us the direct visualization of this universal property. In Fig. 2b we plot the standard deviation *σ* of gaussian fits to all terrace-width probability histograms against the mean step spacing 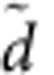. For small *d*, where the gaussian fit works well, a clear linear correlation between 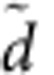 and *σ* is found. This is expected for a repulsive, elastic interaction between steps with constant interaction strength^10^. At large *d* values the probability distribution becomes asymmetric, and the linear correlation is lost. The plot in Fig. 2b suggests that the transition from the elastic to the entropic regime occurs around 
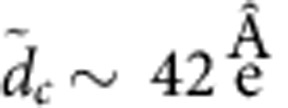
. The *z*-scan in Fig. 2c strikingly images such transition. Here the intensity plot is built with all the terrace width histograms across the curved surface. We also mark with a straight line the center of the gaussian and with the dotted line its width, which we may define, for example, by 
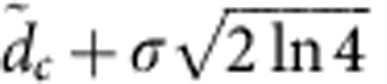
, the point at which the distribution with entropic contributions departs from the purely gaussian curve^12^. As readily visualized in Fig. 2c, beyond *d*=30–50 Å the intensity maximum in the histogram slightly deviates to the left side of the gaussian peak, and a clear ‘tail' develops at large *d* values. Both the leftward shift of the probability maximum and the entropic tail are the basic expectations for steps behaving as one-dimensional, non-interacting fermions^13^, that is, a general property of stepped surfaces with wandering steps in the entropic regime. In the past, the transition from entropic to elastic arrays could only be inferred from data belonging to different samples^12^. Now we can straightforwardly demonstrate it with the use of a (single) curved surface.

### XPS imaging and analysis of the clean surface

**Figure 3:**
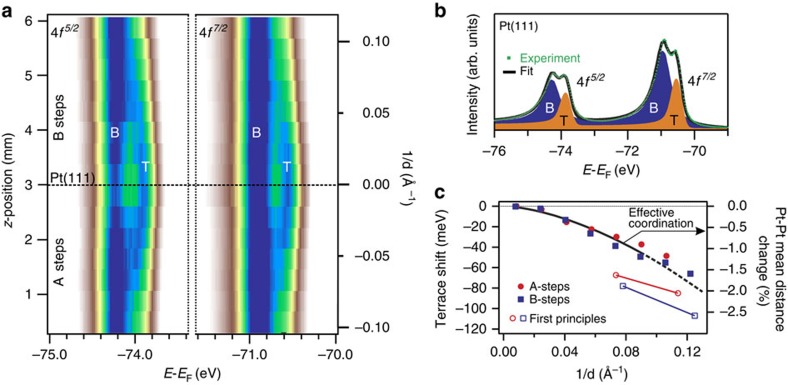
Photoemission scan of the curved Pt(111) surface. (**a**) XPS image showing the evolution of the Pt 4*f* spectrum across the curved crystal. A characteristic step-density-dependent shift is observed in the surface component, revealing a 1/*d*-dependent accumulation of compressive strain at the surface plane. (**b**) Pt 4*f* XPS spectrum corresponding to the (111) plane of the crystal. A least-squares fitting is performed, showing two components, one (B, blue) associated with bulk atoms and another one (T, orange) associated with surface atoms. (**c**) Surface core-level energy variation across the curved crystal. Red and blue open symbols are core-level shifts determined from first-principles calculations for some stepped crystals (Supplementary Fig. 5 and Supplementary Note 2). The black solid line represents the lattice strain with respect to bulk Pt, as deduced from the core-level energy and the associated effective coordination change *n*_eff_(*d*) (see the text).

The transition from the entropic to the elastic regime shown in Fig. 2c reflects the general fact that atomic relaxations in stepped surfaces build up out of step edges, extending inside terraces. Therefore, beyond a critical terrace width 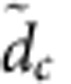, atom-displacement fields from contiguous steps significantly overlap, so as to trigger an effective elastic interaction. X-ray diffraction experiments from stepped Pt(111) prove that step relaxations in fact propagate both towards the bulk and in the terrace, with characteristic decay lengths in the 50 Å range^15^. In average, the Pt stepped surface undergoes, as a function of *d*, structural distortions that affect the topmost planes. Such subtle surface lattice changes are neatly imaged in the Pt 4*f* core-level scan of the curved Pt(111) sample presented in Fig. 3a. The image plot is made of 15 different spectra systematically recorded across the surface. From individual XPS spectra, like the one shown in Fig. 3b, we immediately identify the Pt 4*f*^5/2^ and Pt 4*f*^7/2^ spin–orbit–split lines, both exhibiting separated contributions from the terrace (T) and bulk (B) atoms. Similar results are obtained at other photon energies and measuring geometries, as shown in Supplementary Fig. 3, and analyzed and discussed in Supplementary Fig. 4 and Supplementary Note 1. Taking the center of the crystal as a reference, the *z*-scan image of Fig. 3a allows one to visualize, as a function of *d*, a consistent and symmetric higher binding energy shift in both Pt 4*f*^5/2^ and Pt 4*f*^7/2^ terrace peaks. This is the same subtle energy variation of the surface core-level found in pairs of Rh(111) and W(110) vicinal surfaces^16^,^17^, which the curved sample, with the (111) reference plane in the center, allows to beautifully image and accurately determine.

The shift of the surface core level towards the bulk line shown in Fig. 3a indicates that the surface atomic plane gets compressed as the step density increases, that is, that steps help to release the intrinsic tensile stress of metal surfaces^18^. Such conclusion is also derived from first-principles density functional calculations performed at four different stepped Pt(111) surfaces (Supplementary Fig. 5), as well as from the estimation of the surface-strain derivatives of the Pt 4*f* energy position (Supplementary Note 2). The sign and magnitude of the surface core-level shift in transition metals mostly depends on the degree of filling of the *d*-electron shell, which strongly screens both ion charges and core-hole excitations^19^. For Pt, with an almost filled 5*d*-band, a shift to lower binding energy is expected, and indeed observed, as the Pt 5*d*-band narrows, that is, as the coordination decreases from bulk (*n*=12) to surface (*n*=9), and then to step (*n*=7) (ref. 20). The terrace-size-dependent shift in the surface core-level, imaged in Fig. 3a, can be thus explained within this 5*d*-band picture: the increasing lattice compression results in a larger ‘overlap' of atomic wave functions, that is, in an effective increase in atomic coordination of surface atoms that makes them become more ‘bulk-like'^20^.

A Doniac–Sunjic line fit is systematically performed to all individual XPS spectra of Fig. 3a. We limit the fit to the two B and T lines shown in Fig. 3b. In contrast to the case of vicinal Rh(111) (ref. 16), in Pt(111) one cannot resolve step (*n*=7) or corner (*n*=10, 11) peaks. As discussed in the Supplementary Note 2, this is due to the relative broad line shape of the Pt 4*f* peak (170 meV for T atoms, as deduced from the fit at the center of the crystal). Interestingly, as inferred from the intensity analysis of the fit shown in Supplementary Fig. 4 and discussed in Supplementary Note 1, corner (*C*) atoms contribute to the B peak in Fig. 3a, whereas step (*S*) emission is part of the T peak. The Doniac–Sunjic fit determines the energy shift Δ*E*_T_(*d)* of the surface core level in the most accurate way, because the Pt(111) reference is present in the same sample. The resulting data points are shown in Fig. 3c and compared to first-principles calculations (open markers) performed for two *d* values at each type of step (Supplementary Fig. 5). The latter give the correct sign, but overestimate the measured shift, particularly at large *d*, although the ultimate reason for this overestimation is not clear (Supplementary Note 2).

From Δ*E*_T_(*d*) we can obtain an estimate of the average nearest-neighbor distance variation (defined as strain in the discussion below) in surface atoms (*n*=9), applying the ‘effective coordination' *n*_eff_ concept^20^,^21^. Details are given in the Supplementary Note 3. In short, a *d*-dependent effective coordination *n*_eff_(*d*) is derived from Δ*E*_T_(*d*), assuming the experimental core-level shift of −400 meV from surface (*n*=9) to bulk (*n*=12). Then, the average strain Δ*R*(*d*)=*R*_bulk_−*R*_surface_(*d*), is given by *n*_eff_(*d*)=9 × *e*^−*b*Δ*R*(*d*)^, where the constant *b* refers to the exponential decay length of the atomic charge density distribution in Pt (*b*=1.27 Å^−1^, see ref. 22). Figure 3c represents the measured surface core-level energy variation, together with the deduced strain at the surface Δ*R*(*d*). This reaches a ∼2% reduction of the average nearest-neighbour Pt-Pt distance at the highly stepped Pt(335) and Pt(355) planes, in fair agreement with X-ray diffraction data^15^,^23^. The average compression of surface atoms is also clear from the theoretical *ab initio* analysis. The theory gives the same order of magnitude, but a smaller reduction in the average strain at stepped planes, as compared with the ‘effective coordination' model (Supplementary Note 3).

### XPS imaging and analysis of the CO-covered surface

**Figure 4:**
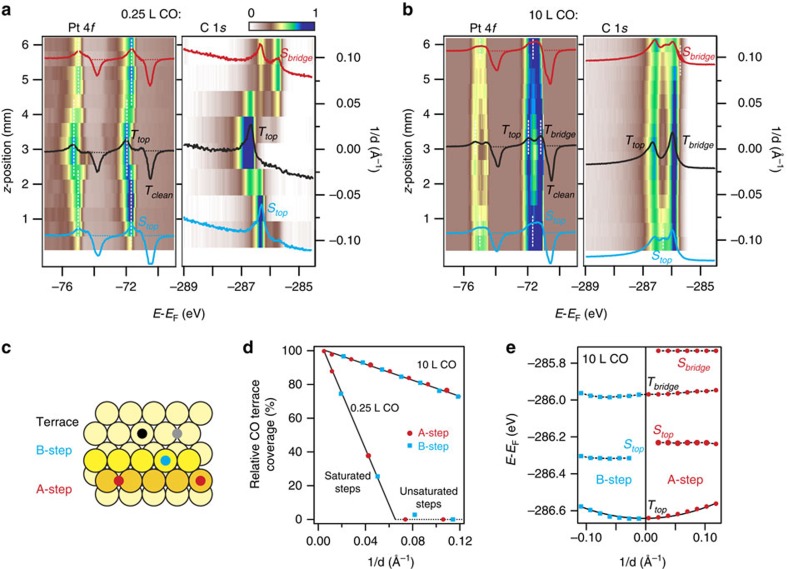
Photoemission scans of the CO-adsorbed Pt(111) curved surface. Pt 4*f* and C 1*s* images of the curved surface after dosing with (**a**) 0.25 L and (**b**) 10 L of CO at 300 K, respectively. The Pt 4*f* image represents the intensity difference with respect to the clean surface spectrum (4*f*_CO_–4*f*_clean_). Profiles at the (111), (335) and (553) crystal orientations appear overlaid. (**c**) Sketch of the different chemisorption sites for CO on Pt(111), which are detected in panels (**a**,**b**). (**d**) Peak amplitude variations for the total C 1*s* terrace emission (*T*_top_+*T*_bridge_) relative to the total C 1*s* emission at the Pt(111) surface (center of the crystal). The linear decrease reflects the increasing presence of CO-saturated steps at both low and high-dose regimes. (**e**) Binding energy variation across the curved surface for all C 1*s* components. The 1/*d* dependence for *T*_top_ reflects an unexpected chemisorption energy variation for CO at stepped surfaces.

The Pt(111) surface has been a model platform to study CO chemisorption, around which many controversies arose in the past, for example, the preferred adsorption sites on terraces^24^. The catalytic activity of Pt steps is still matter of discussion, for example, the existence of an optimal step density to promote CO oxidation under realistic, high-pressure conditions^25^. The chemisorption of CO on stepped Pt(111) is known to exhibit a rich hierarchy of sites. The curved surface approach allows one to reveal such hierarchy in single C 1*s* and Pt 4*f z*-scans, shown in Fig. 4a,b. The images correspond to two radically different CO adsorption stages, namely far-below (0.25 L) and well-above (10 L) the Pt(111) saturation dose (∼3 L) at 300 K (ref. 26). With 0.25 L (∼0.08 CO monolayers), we readily identify the sequence of preferred adsorption places, whereas the map of the saturated surface allows us to quantify all available adsorption sites at 300 K and ultra high vacuum (UHV) conditions. For Pt 4*f* we represent the adsorbed-clean difference spectrum, to enhance CO-adsorbed satellites. The Pt 4*f* and C 1*s* XPS spectra at characteristic sample orientations, as well as line fit analysis details and uptake experiments (to determine the correspondence between CO dose in L and coverage in monolayer), are shown in detail in Supplementary Figs 6 and 7. The different adsorption places are sketched in Fig. 4c, corresponding to on-top *T*_top_ and bridge *T*_bridge_ on terraces, and on-top *S*_top_ and bridge *S*_bridge_ at steps. C 1*s* images nicely reflect the chemisorption asymmetry for A-type (*S*_top_ and *S*_bridge_) and B-type (only *S*_top_) steps^27^, which is also reproduced by our theory (Supplementary Note 4). From the C 1*s* scans the known hierarchy of occupation of surface sites is evident, namely the step-over-terrace preference in Fig. 4a (rapid quenching of terrace *T*_top_ signal in parallel to the growth of *S*_top_ and *S*_bridge_ peaks away from the sample centre), and the *T*_top_ versus *T*_bridge_ adsorption in Fig. 4b.

The potential of the XPS scanning of the CO-covered Pt curved surface goes beyond the mere visualization of the chemisorption site preference, allowing the accurate quantification of surface-orientation properties. First of all is the CO sticking probability. Although Fig. 4a,b demonstrate that all adsorption sites are distinctly occupied as a function of 1/*d*, it is interesting to remark that the total C 1*s* intensity remains constant across the curved surface. This means that CO molecules are redistributed among different adsorption sites, and between steps and terraces, but the absolute coverage, at any CO dose, does not depend on *d*, that is, the CO sticking probability is the same for all surface planes. This contrasts with the variable sticking probability of other gases, such as O_2_ (ref. 4). On the other hand, by performing Doniac–Sunjic fits to C 1*s* spectra we can safely separate terrace and step contributions and study the flow of CO from terraces to steps. The normalized terrace C 1*s* intensity *T* is plotted in Fig. 4d. At the very low dose, the T signal drops linearly as a function of 1/*d*, up to 1/*d*_0_∼0.06 Å^−1^, at which the terrace *T*_top_ emission vanishes. At the saturation dose, the linear drop of T is somewhat larger for B steps. The drop in *T* away from the (111) surface always reflect the same property, namely a local saturation that occurs, first, at steps, and then, at terraces. The curved surface experiment allows us to accurately determine the room temperature CO-saturation value at both regimes. In the low dose experiment with *θ*_CO_=0.08 monolayers (ML) and *d*_0_=15 Å, and assuming that steps extend a single atomic row *a*_*row*_=2.4 Å inside the terrace, it is easy to deduce the density of (one-dimensional) adsorption sites at saturated steps as *θ*_CO_ × *d*_0_/*a*_row_=0.50 ML (ref. 27). For the high-dose experiment straight lines fit the data for a slightly lower 0.47 ML saturation at both steps and terraces alike.

C 1*s* core-level energies for all adsorption sites at CO saturation are plotted in Fig. 4e. Interestingly, energies, which agree with previous literature values and first-principles calculations^2^,^26^,^27^,^28^,^29^, do not vary with 1/*d*, except *T*_top_. This is again a very subtle effect (60 meV shift from the sample center to the edges), falling beyond the limits of the XPS accuracy if two different samples were to be compared^2^, such that only the C 1*s z*-scan on the curved surface allows one to unveil. One may be tempted to connect this *d*-dependent *T*_top_ shift with the increasing surface lattice compression across the curved Pt substrate, probed in Fig. 3. However, a direct correlation can be discarded, since we predict a C 1*s* shift to higher binding energy for a CO molecule adsorbed on a compressed Pt(111) terrace (Supplementary Note 4). In reality, it is known that CO saturation induces a sizeable compressive stress on Pt(111), as a result of a rather complex (and poorly known) interplay between substrate/adsorbate and adsorbate/adsorbate interactions^18^. Moreover, such CO-induced compressive stress largely compensates the intrinsic tensile stress of clean Pt(111) (ref. 18). Thus, stress-release, for example, by steps, is expected to work in opposite directions in clean and CO-covered Pt(111), and that is respectively reflected in the negative Pt 4*f* (Fig. 3a) and the positive *T*_top_ C 1*s* (Fig. 4e) step-dependent, core-level shifts.

## Discussion

The use of properly designed curved crystals opens research frontiers to investigate surface chemistry and growth problems where steps are involved. Scanning the curved surface (*z*-scanning) with standard probes (LEED, STM and XPS) allows the imaging of very subtle *d*-dependent properties, which may be critical to physical-chemical processes. Here we have demonstrated the full potential of this approach with a Pt(111) curved crystal. We revealed a 1/*d*-dependent accumulation of compressive strain in stepped surfaces, visually manifested in the *z*-stack of STM statistical histograms, and quantitatively estimated through the *z*-scan of surface core-levels in XPS. Additionally we have demonstrated the power of the approach to study the model CO/Pt(111) system in simple terms. The curved sample allowed us to straightforwardly determine the hierarchy, and even the density of different adsorption sites with a single C 1*s* core-level *z*-image. This ability can have enormous importance to study the catalytic activity of steps with XPS under real pressure conditions, for example, by imaging the active catalytic phases nucleated at step edges^25^,^30^.

## Methods

### Curved sample processing in ultra high vacuum

The curved Pt(111) surface (Bihurcrystal Ltd., Spain, www.bihurcrystal.com) is obtained by mechanical erosion of a flat Pt(111) crystal, followed by mechanical polishing down to 0.25 μm grinding. Sputtering-annealing cycles of the crystal are performed in the usual way for metal surfaces, that is, 1-keV Ar ion beam energy, in grazing incidence and parallel to surface steps, plus 5 min annealing to 800 °C. The reduced size (6 × 6 mm^2^) of the sample and its shallow curvature ensures the homogeneous surface cleaning and crystalline perfection. The structural analysis performed by LEED and STM shows no macroscopic alterations in the curvature of the crystal after extensive sputter-annealing treatments.

### STM imaging and analysis

STM images have been systematically recorded using a variable temperature STM set-up (Omicron). The coarse movement of the STM piezos allows us to access different Pt surface orientations on the curved crystal. To image the curved surface we used a tunnelling current of ∼0.1 nA and a sample bias of ∼−1 or ∼+1.1 V. The analytical process of the STM images has been explained in detail elsewhere^11^. In summary, we carry out a thorough analysis of individual frames with sizes between 40 × 40 and 300 × 300 nm^2^, depending on the terrace width, using the WSXM software^31^. The STM analysis is always limited to surface areas exhibiting homogeneous step arrays in the μm scale. STM images are automatically processed, and a probability histogram is then produced. Finally, a gaussian fit is applied to the data, which gives the value 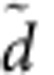 in each image, as well as the standard deviation *σ* defined as 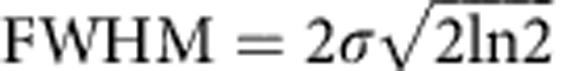, FWHM being the full width at half maximum of the gaussian probability curve^11^. STM experiments were carried out at 300 K.

### X-ray photoemission experiments

High-resolution XPS experiments were performed at beam line I311 at MAX II in Lund, Sweden. To achieve accurate *z*-scans across the curved sample, the light spot size was reduced to 100 μm by means of the exit slit. A *p*-polarized grazing incident geometry was employed, with the photoemission plane parallel to the surface steps (perpendicular to the *z*-scanning direction). Such measuring geometry minimizes the spread of the beam over the curved surface, while reducing the XPS *z*-scan procedure to a simple sample translation. We estimate a probing miscut range on the sample Δ*α*<0.25°, which in reality corresponds to the same surface orientation accuracy commonly achieved in flat crystal samples^11^. XPS *z*-scans were taken with the sample estimated temperature in the range of 150–200 K. The photon energy *hν* was varied to optimize cross section, surface sensitivity and, in the case of Pt 4*f*, the ability to resolve surface and bulk emission lines across the whole curved surface. We recorded individual XPS spectra at 15 different *z*-positions (0.35 mm steps). The clean sample was annealed above 400 K after every *z*-position spectrum, in order to avoid CO accumulation during each measurement step (∼10 min per *z*-position).

### First-principles calculations

Density functional theory calculations were performed in order to test the connection between surface strain and core-level shifts, as well as to explore CO adsorption on several stepped Pt surfaces. We used the Vienna *ab initio* simulation package (VASP)^32^ with the Perdew–Burke–Ernzerhof functional^33^, the projected-augmented-wave method, and a plane-wave cutoff of 230 eV for clean Pt surfaces and 400 eV when CO was adsorbed on the surface. We studied the Pt(111), Pt(557), Pt(332), Pt(335) and Pt(221) surfaces using slabs containing five atom layers. The two upmost layers were relaxed. The number of k-points was always consistent with a 15 × 15 sampling of the surface Brillouin zone of Pt(111). The core-level shifts (CLS) were calculated in the so-called final state approximation^34^. Similar results for the variation of the Pt 4*f* surface CLS with strain/miscut angle were found using the initial^34^ and the transition state^35^,^36^ approximations. We also checked our results using supercells containing different number of atoms and found typically small differences (for example, ∼20 meV for the Pt 4*f* CLS of surface atoms in Pt(111) computed using a 2 × 2 and a 4 × 4 supercell). In order to compare the CLSs from different vicinal surfaces (corresponding to supercells of different sizes) we computed the CLS of the 4*f* Pt level of terrace and step atoms with respect to bulk atoms. Thus, in each calculation we defined the bulk atoms as those belonging to the central layer of the slab (we only compared results obtained with slabs of the same thickness). The average over inequivalent atoms in the central layer of each slab added a ∼10 meV uncertainty to the computed CLSs.

## Additional information

**How to cite this article:** Walter, A. L. *et al*. X-ray photoemission analysis of clean and carbon monoxide-chemisorbed platinum(111) stepped surfaces using a curved crystal. *Nat. Commun.* 6:8903 doi: 10.1038/ncomms9903 (2015).

## Supplementary Material

Supplementary Information Supplementary Figures 1-7, Supplementary Notes 1-4 and Supplementary References
