# Supplementary material for: X-ray photoemission analysis of clean and carbon monoxide-chemisorbed platinum(111) stepped surfaces using a curved crystal
Source: Nat Commun. 2015 Nov 12;6:8903. doi: 10.1038/ncomms9903 (PMC4660355; doi:10.1038/ncomms9903)
Supplement: Supplementary Information — Supplementary Figures 1-7, Supplementary Notes 1-4 and Supplementary References [file ncomms9903-s1.pdf]

Supplementary Information

Supplementary Figures

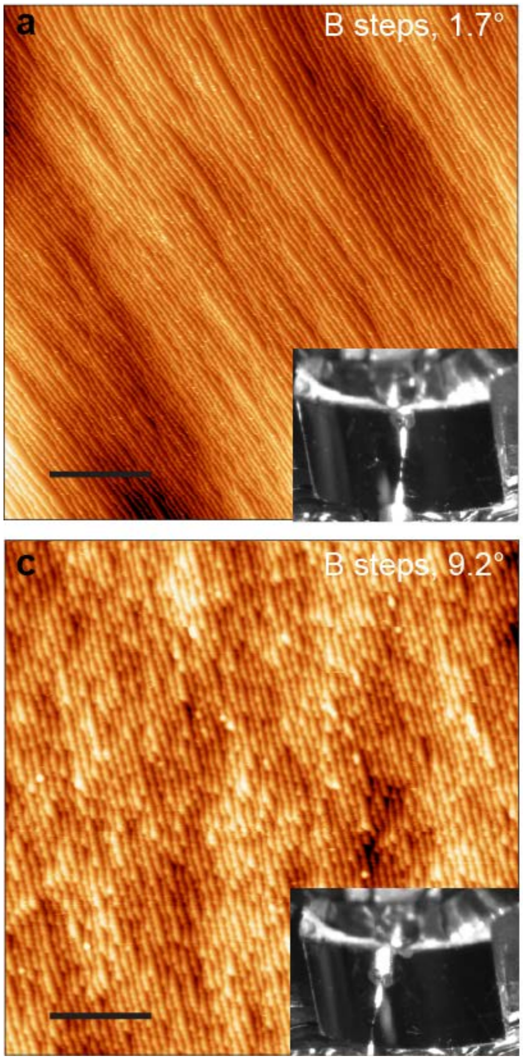

Supplementary Figure 1. Large area scans by Scanning Tunneling Microscopy. STM images showing large areas of the Pt(111) curved surface at both A and B sides of the crystal. The miscut angle α and the step type A or B appear indicated in each case. The quality of the sample is demonstrated by the homogeneity and regularity of the step array (generally with α >1.5°). From such homogeneous areas, smaller frames are selected to perform the statistical analysis of the terrace width (see Supplementary Fig. 2). The *z* position is chosen by guiding the STM tip with a video camera, as shown in the insets. The scale bars correspond to: 140 nm in (**a**), 100 nm in (**b**), 20 nm in (**c**) and 60 nm in (**d**).

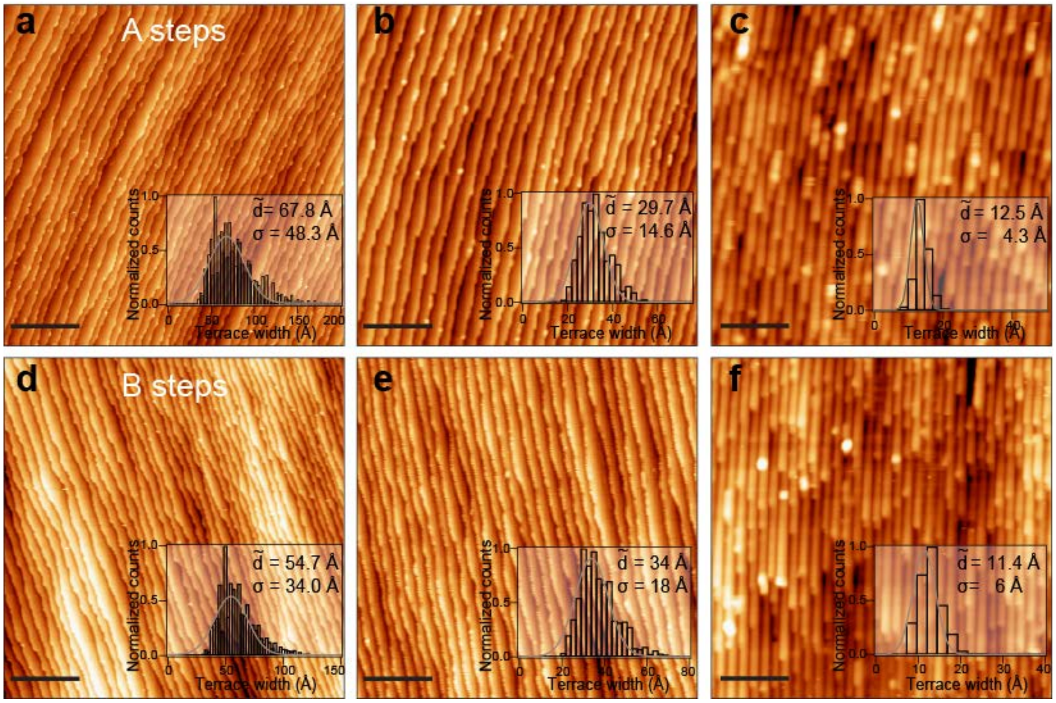

Supplementary Figure 2. Statistical analysis of STM images. Sequence of STM images across the curved Pt(111) surface, and their corresponding terrace-width probability histogram. Overlaid is shown the Gaussian fit of the histogram in each case. The resulting mean terrace width $\tilde{d}$ and standard deviation σ are plotted in the *1/d* plot of Fig. 2 in the main text. The scale bars correspond to: 50 nm in (**a**) and (**d**), 20 nm in (**b**) and (**e**), 8 nm in (**c**) and (**f**).

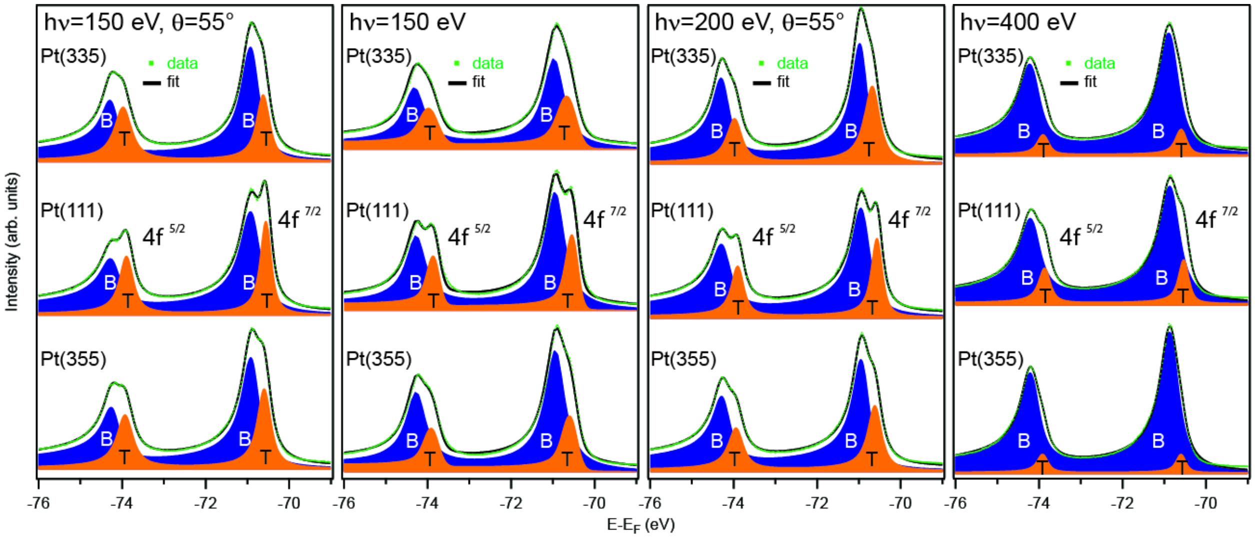

Supplementary Figure 3. Pt 4f XPS spectra on clean curved Pt(111) crystal. XPS spectra with their corresponding fitting lines for the Pt 4f doublet, at three different sample locations, and under different measuring conditions photon energy $h\nu$ and emission angle θ. Two features, namely surface (mainly terrace intensity T and bulk B are clearly visible.

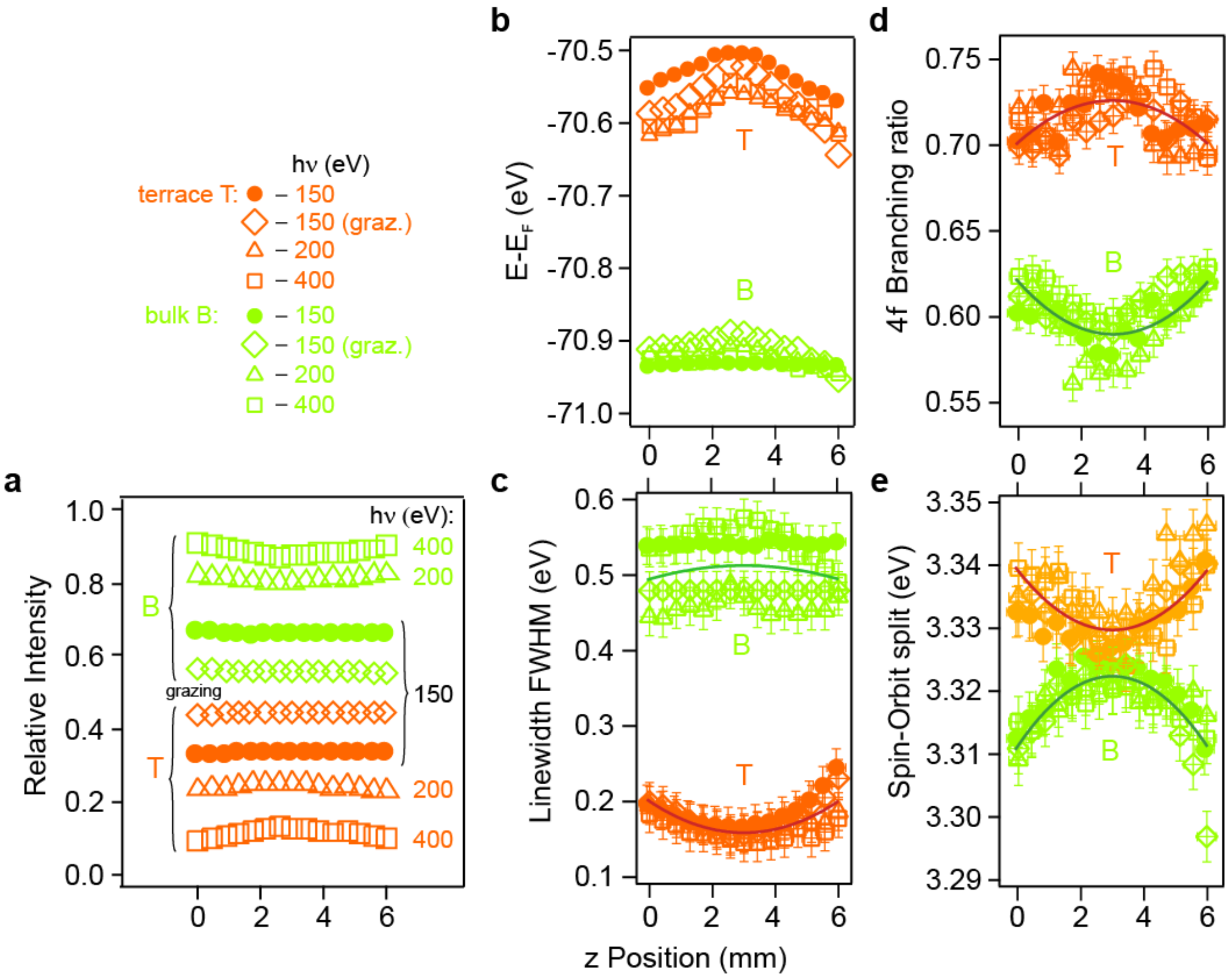

Supplementary Figure 4. Influence of measuring conditions on XPS spectra line fit. Evolution of the main fitting parameters (peak amplitude (**a**), energy (**b**), and line width (**c**), with background and asymmetry fixed for all *z*-positions) across the curved sample for 150 eV, 200 eV and 400 eV photon energies, and the normal and grazing emission angles for $h\nu$=150 eV. Data shown correspond to the 4f $^{7/2}$ peak of Supplementary Fig. 3. The lines in (**c**) mark the average line width of both *B* and *T*. (**d-e**) Evolution of the 4f $^{5/2}$-4f $^{7/2}$ spin-orbit-splitting energy and branching (amplitude) ratio across the curved surface. The lines fit all data at once. Changes are expected due to the *1/d* increase of strain at a stepped surface.

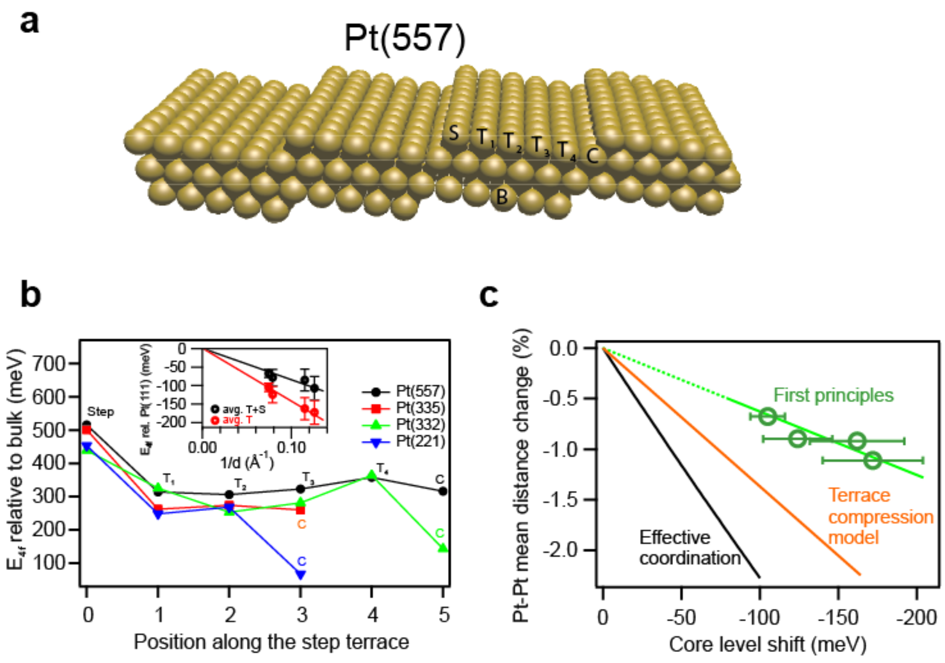

Supplementary Figure 5. Calculated Pt 4f core-level-shift (CLS) as a function of the miscut angle and surface strain. (**a**) Drawing of the Pt(557) stepped surface. Indicated are the differently coordinated atoms: step *S* (*n*=7), corner *C* (*n*=10), and bulk *B* (*n*=12), T_i stand for terrace atoms (*n*=9) successively numbered from the step edge to the corner. (**b**) Pt 4f core-level energy of *S* and T_i atoms with respect to the bulk atom *B*, calculated for different stepped surfaces and different atoms along the terrace. The inset shows the resulting CLS with respect to the flat Pt(111) surface, after averaging over all the atoms in a given terrace (*T*, red), or additionally considering step atoms (*T*+*S*, black)], as a function of 1/*d*. The CLS diminishes as the miscut angle increases, as measured by XPS. Continuous lines in the inset are linear fits. (**c**) Average nearest-neighbor distance change of *T* atoms as a function of the core-level shift, as determined from the *effective coordination* model (black line, described below in Supplementary Note 3), from a model based on a DFT analysis of core-level positions in an artificially compressed Pt(557) terrace (orange line, described below in Supplementary Note 2 and referred to as *terrace compression model*), and from the first-principles calculation for the different surfaces of (**b**) (symbols). The green line is a guide to the eye for the latter. Errors bars in the inset of panel (**b**) and panel (**c**) reflect the uncertainty associated with the bulk reference atom plus the uncertainties in the calculation of the surface CLS in Pt(111). The green data in panel (**c**) are the same as those presented in red in the inset of panel (**b**).

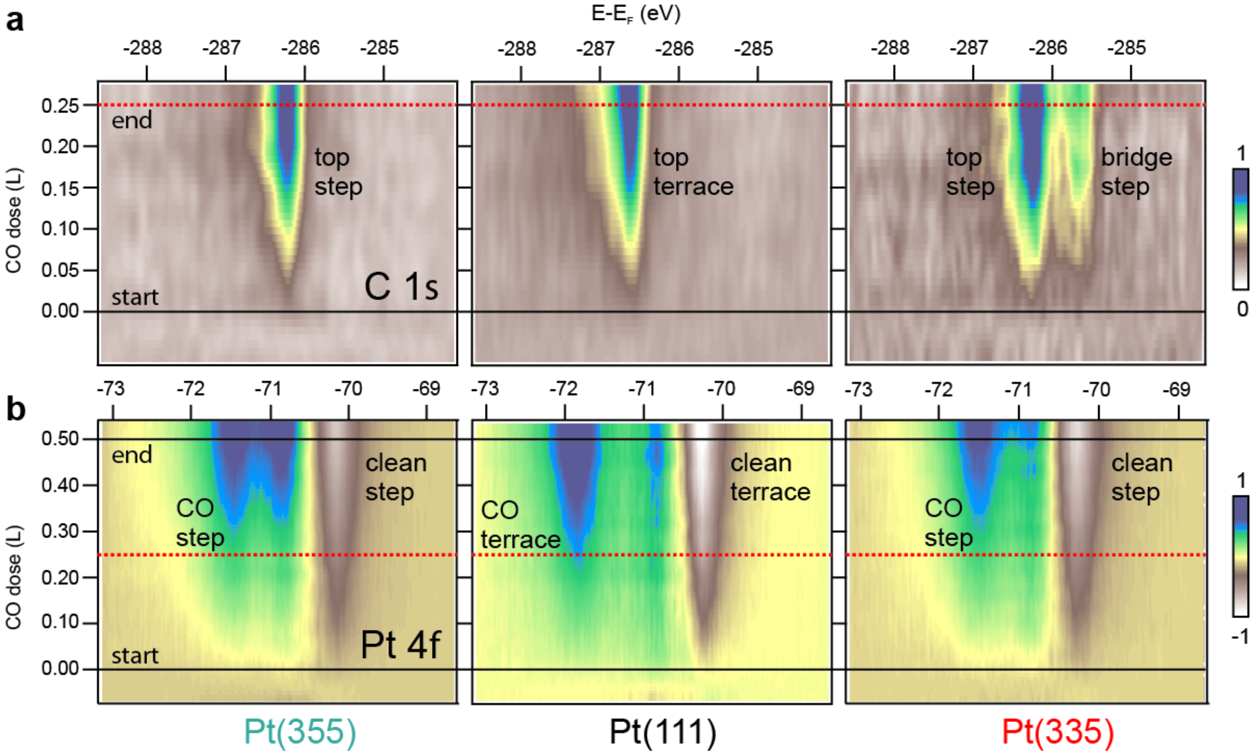

Supplementary Figure 6. Hierarchy of CO chemisorption on curved Pt(111). Image of the CO uptake at 300K measured on (**a**) the C 1s (up to 0.25 Langmuir (L)) and (**b**) the Pt 4f $^{7/2}$ core levels (up to 0.5 L), and, from left to right, at the (355), (111) and (335) planes of the curved Pt(111) crystal.

The C 1s intensity evolution shows the successive appearance of distinct lines for CO adsorbed at the indicated sites that have been identified in earlier works: on-top adsorption at (111) terraces for the flat surface at -286.63 eV electron energy [1, 2], step on-top (-286.35 eV) for B-type steps [2, 3], and both step on-top and bridge (-285.73 eV) for A-type steps [4]. The bridge adsorption on terraces is detected only with higher CO doses at -286.97 eV (see Supplementary Fig. 7), and it also coincides with literature observations [3]. In Fig. 4 **c** of the main text we sketch all different adsorption sites.

The Pt 4f plot represents the difference between the clean surface peak and the CO-covered one $4f_{CO}$-$4f_{clean}$, which is taken as the surface is progressively exposed to CO. This allows highlighting the variation in the Pt 4f peak upon CO adsorption. At this low exposure range, two clear growing features are detected, one for terraces at the Pt(111) plane (terrace on-top at -71.65 eV [1, 5]), and another one for steps at both Pt(335) and Pt(355) faces (step on-top at -71.44 eV for B-type steps [5]). Also some lower intensity arises close to the clean bulk emission, due to terrace-bridge adsorption (as detected in Ref. [5], and clearly visible at the higher 10 L dose in Supplementary Fig. 7).

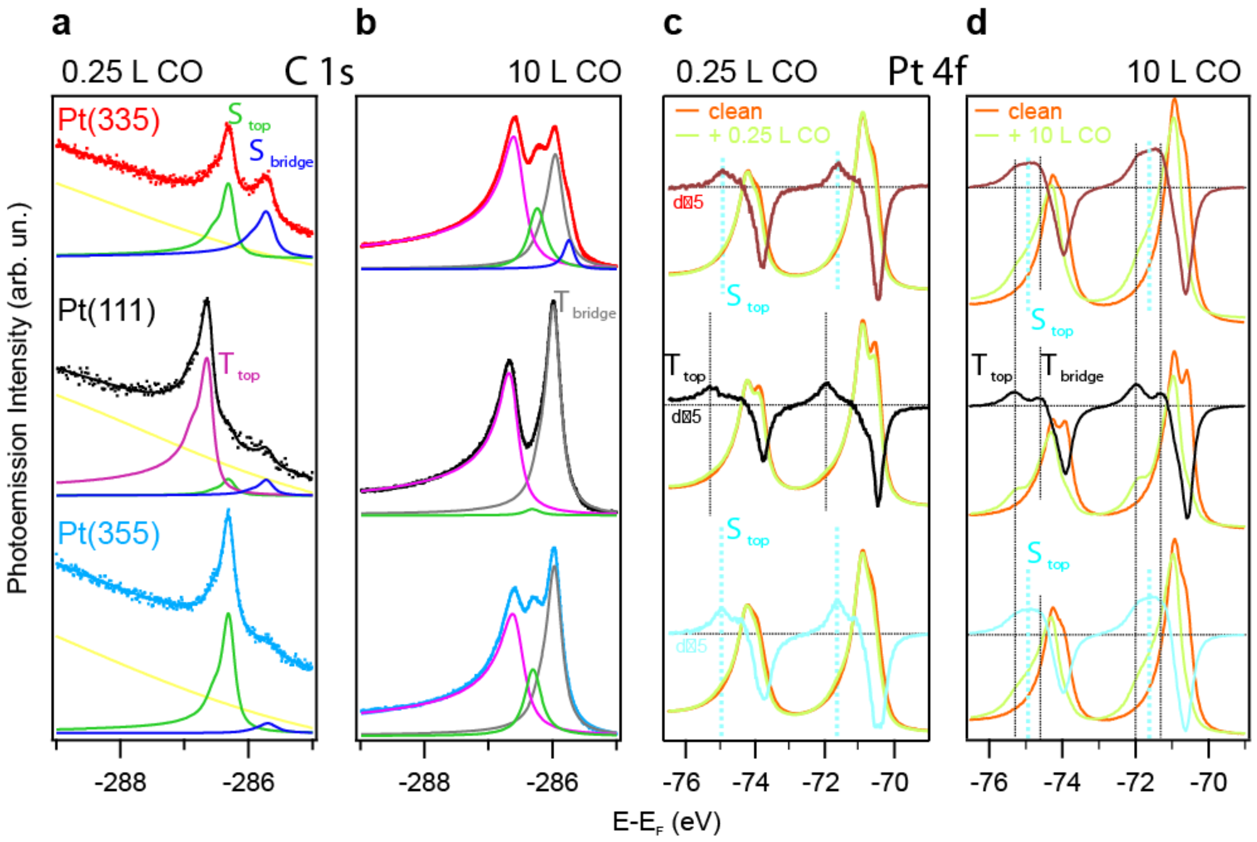

Supplementary Figure 7. High resolution core level spectra as a function of CO coverage. (**a-b**) C 1s and (**c-d**) Pt 4f core level spectra for (355), (111) and (335) planes of the curved Pt(111) crystal, taken with low CO dose (0.25 L) and upon surface saturation (10 L), as indicated. For C 1s, we also include the functional lines derived from least-squares, Donjac-Sunjic line-fits, utilizing a number of peaks that equals the number of observed features. Such a line-fit is performed all across the curved surface. The resulting amplitudes and peak positions are analyzed in Fig. 4 in the main text. For Pt 4f we compared the CO-covered spectrum with the clean surface one, and include the resulting difference $4f_{CO}$-$4f_{clean}$. This helps to identify distinct, new features arising from CO-adsorbed Pt atoms (ticks and lines).

Supplementary Notes

Supplementary Note 1: Photon energy dependence of XPS spectra and line fit analysis

The characteristic results for three different *z* values taken at different photon energies ($h\nu$ from 150 eV, to 200 eV, and 400 eV) and emission angles (θ=0° at normal emission and θ=55° grazing) are shown in Supplementary Fig. 3). Doniac-Sunjic line fits were systematically performed to both Pt 4f $^{5/2}$ and Pt 4f $^{7/2}$ lines at once. We limited the number of fitting peaks to the clearly visible features, namely a bulk peak *B* and a surface peak *T* for both Pt 4f $^{5/2}$ and Pt 4f $^{7/2}$ peaks. The Pt 4f core-level does not allow to resolve step *S* and corner *C* atom emission (see Fig. 1 **b** in main text), as in shallow core-levels of Rh and W [6, 7]. Although grazing incidence provides better surface sensitivity (*T*/*B* amplitude), we observe a rather inhomogeneous variation at the highly stepped edges of the crystal, reflected in the fit results displayed in Supplementary Fig. 4. Thus, for Pt 4f, we found the best and more reliable measuring conditions at $h\nu$=150 eV and normal emission, i.e., parallel to the [111] crystal direction.

By examining how the measuring conditions ($h\nu$ and θ) influence the fit results (Supplementary Fig. 4 **a-c**), we find that the peak energies keep the same values, within error bars, for all $h\nu$ and θ values (Supplementary Fig. 4 **b**). Thus, the same 1/*d*-dependent shift of the *T* peak as referred to the bulk peak can be observed in all cases. This was explained in the main text as due to accumulation of compressive strain (Fig. 3).

For the *T* peak the line width variation (Supplementary Fig. 4 **c**), as demonstrated through the first principles calculations discussed in Supplementary Note 2, is likely related to the variation of binding energy expected for terrace atoms as a function of the distance to the step edge. Due to the natural line width of the *T* atom emission (170 meV, as measured at the center of the sample), emission from different atoms at terraces cannot be resolved, nor the emission from the *S* atom. Thus, all contributions, including *S* atom emission, add up to a single *T* line of increasing width. As a way of example, for Pt(223) with 5 atom wide terraces the calculation gives a smooth variation of binding energy from *S* to *T* atoms of about 200 meV, which results in a single peak of increasing width from 170 meV at the center of the terrace to 260 meV at the Pt(223) plane. This agrees with the experimentally found linewidth variation of the *T* peak shown in Supplementary Fig. 4 **c**, which changes from 170 meV at the Pt(111) plane to 230 meV at the Pt(223) plane.

As compared with the *T* peak, the *B* peak exhibits an even larger variation in the fitting parameters, namely energy and particularly line width, among the data sets in Supplementary Figs. 4 **b** and **c**. This is a clear signature of the importance of photoelectron diffraction (PED) effects, which mostly affect bulk emission. PED effects combined with the significant low energy tail observed (i.e., the large asymmetry of the *B* peak), pose limits to a detailed quantitative analysis based on the peak amplitudes, particularly at the stepped sides of the crystal, where *T* and *B* peaks get close together.

Assuming such limitations, the fit still reveals two remarkable properties in all data sets:

(i) the steady lowering of the surface/bulk (*T*/*B*) peak amplitude ratio as a function of 1/*d*, directly visible in Supplementary Fig. 4 **a**. We attribute this effect to the contribution of corner atoms (marked as *C* in Fig. 1 **b** in the main text) to the *B* peak. *C* atoms have larger coordination (10 and 11, for A-type and B-type steps, respectively) than step (7) or terrace (9) atoms, and hence their core level is found between *T* and *B* peaks [6]. Note in the sketch of Fig. 4 **c** (main text) that steps leave *C* atoms partially uncovered, nominally 2/3 of an atomic row in A steps and 1/3 in B steps. Thus the increasing presence of *C* atoms as

a function of 1/*d* will result in an effective "transfer" of intensity from the right side (*T* peak) to the left side (*B* peak) in both 4f $^{5/2}$ and 4f $^{7/2}$ peaks. One may readily estimate such intensity evolution by assuming a simple exponential attenuation scenario for core-level intensities of subsurface atoms. However, the effect found, although consistent with observation, is very subtle (10-20% depending on photon energy and emission angle), i.e., within the limits set by PED effects.

(ii) The step-dependent evolution of the line fit to the Pt 4f line also reveals a 1/*d*-dependent change in magnitude and branching ratio of the 4f$^{5/2}$-4f$^{7/2}$ spin-orbit splitting in all data sets. This is shown in the right panels of Supplementary Fig. 4 **b**. Within very minor variations, all data show the same values for *T* and *B* peaks and the same 1/*d* tendency, which allows us to discard PED or final-state cross section effects. The thick line fits all data sets at once to average out all such effects. In average, for *T* the branching ratio is close to the expected degeneracy of each spin state (0.75), but the *B* peak notably departs from such value. The latter agrees with similar observations made for the Au 4f bulk peak at relatively low photon energies [8]. But the remarkable observation is the variation of both the branching ratio and the splitting energy in opposite directions for *T* and *B* peaks. Again the variable strain across the curved plane is invoked to explain such effect.

Supplementary Note 2: First principles calculation of the surface core-level-shift of 4f Pt as a function of the miscut angle and surface strain

Supplementary Figure 5 **b** shows the computed shift of the 4f Pt core-level with respect to the bulk reference for different atoms in the vicinal surfaces studied here. The energy of the Pt 4f core-level strongly depends on the atom coordination. For step (*S*) atoms it is maximal and largely independent on the miscut angle, although it shows a clear dependence on the type of step: ~500 meV for type-A steps and ~450 meV for B steps. Following the nominal atom coordination, corner (*C*) atoms display the smallest CLS, with a very strong dependence on the step type. In contrast, the CLS of terrace (*T*) atoms does not show a clear dependence on the step orientation. In this case we find a stronger dependence on the miscut angle and the position of the atom within the step terrace. As shown in Supplementary Fig. 5 **b**, the average CLS of the *T* atoms tends to diminish as the miscut angle increases in agreement with our experimental observations.

The dependence on the miscut angle is more clearly presented in the inset of the Supplementary Fig. 5 (**b**), where we plot the average energy of the Pt 4f level, relative to that of surface atoms in flat Pt(111) (computed value 430 ± 7 meV, in excellent agreement with experiment), for T_i atoms (black symbols) and T_i and *S* atoms (red symbols) as a function of *1/d*, with *d* being the width of the terraces in each surface. In the experiment, due to the natural width of the Pt 4f T atom peak (170 meV, see Supplementary Fig. 3), *S* and *T* atom emissions cannot be separately resolved. For this reason, we compare our experimental results to the *T*+*S* average. In agreement with experiment, the Pt 4f level shifts down as a function of the miscut angle. However, data from first-principles density functional calculations overestimate the effect by more than 50%. The reason for this large overestimation is not clear at present and might be simply related to the limitations of some of the approximations utilized (e.g., use of semilocal density functionals or the lack of core relaxation in the current implementation of the final state approximation within VASP [9]).

According to our interpretation, this shift is primarily due to the average compressive strain present in the terraces of the vicinal surfaces. An analysis of our relaxed geometries confirms that a substantial

compressive strain exists in the terraces and that it increases with increasing miscut angle [e.g., the in-plane compression of the terraces goes from -2.76 % for Pt(557) to -4.97 % for Pt(221)]. Interestingly, the in-plane strain is somewhat larger for the terraces of type-B steps than for A steps. Green data in Supplementary Fig. 5 **c** depict the compression of the average distance to the nearest neighbors for surface Pt atoms as a function of the shift of the 4f level in the different vicinal surfaces. In spite of the large relative uncertainties of the computed CLS's, data suggest a clear correlation between both observables, strain and core level energy.

To avoid the obvious limitations imposed by performing explicit supercell calculations, as well as to explore in more detail the connection between the surface strain and the CLS, we have also followed an alternative route. In brief, applying different deformations around the relaxed structures of the Pt(557) and Pt(111) surfaces we can estimate the variation of the 4f Pt CLS as a function of the in-plane and out-of-plane applied strains. Additionally, analyzing the relaxed geometries of the studied stepped surfaces we can find the relation between the average in-plane and out-of-plane strains in the terrace. Assuming that this approximate relation holds for terraces of all sizes, we can express the variation of Pt 4f level as a function of the average Pt-Pt distance change in vicinal surfaces. The corresponding data are plotted in Supplementary Fig. 5 **c** (orange solid line) and labelled *terrace compression* model. This model also gives a clear shift of the Pt 4f level to higher binding energies as the compressive strain in the terrace increases. Interestingly, the data of the model are much closer to those deduced from the experiment using the empirical *effective coordination model* (see Supplementary Note 3), giving CLS's significantly smaller than those deduced from comparing explicit calculations for different stepped surfaces. In summary, given the numerical uncertainties in the CLS calculations and the small size of the shifts we are addressing, we believe that our first-principles calculations give a reasonable account of the experiments and confirm that the change of the strain of the surface layer as a function of the miscut angle is the main source of the observed effect.

Supplementary Note 3: Effective Coordination model: Empirical surface-strain from core-level shift

XPS straightforwardly reveals the chemical composition of a surface, although it is particularly unique to determine changes in the atomic environment through the core-level energy shifts. In particular a characteristic shift is observed when the atomic coordination *n* is reduced from the bulk to the surface and to the step edge [6, 7]. For Pt, Bianchettin *et al.* have experimentally found a linear relation between the coordination number *n* and the core-level energy [7]. This is indeed expected from simple atomic wave function overlap arguments. Moreover, Bianchettin *et al.* showed that deviations from the pure proportionality are well-explained as due to Pt-Pt bond relaxations, and not to core-hole screening changes, which appear to have very little influence. Bianchettin *et al.* defined an effective coordination number n_{eff}, by weighting the actual length of each individual Pt atom bond R_j as:

$$n_{eff} = \sum_j e^{b(R_{bulk}-R_j)} \quad (1)$$

where the sum runs for all *j* nearest neighbors. *b* stands for the decay constant calculated for the charge density of an isolated Pt atom, which in the range of the interatomic distances (R_{bulk}=2.77 Å) may be taken as *b*=1.27 $Å^{-1}$ [10]. Considering the Pt(111) surface (*n*=9), and the average change in lattice

constant with respect to the bulk (strain) as ΔR=(R_{bulk}-$R_{surface}$)/R_{bulk}, we can derive the effective coordination at the surface as a function of lattice strain as:

$$n_{eff} = 9 \times e^{1.27 \times 2.77 \times \Delta R(d)} \quad (2)$$

Next we consider the surface core level shift $\Delta E_{9\rightarrow 12}$ = -400 meV for Pt 4f, as shown in Fig. 3 **b**, and assume a linear variation of the core-level energy as a function of n_{eff} between n=9 and n=12 as ΔE = -400 × (n_{eff}-9)/3 [meV]. By inserting this expression into Eq. 2, we can thus derive the strain as a function of the terrace size *ΔR(d)* as:

$$\Delta R(d) = -\frac{1}{3.52} \times ln\left[\frac{\Delta E(d)}{1.2} + 1\right] \quad (3)$$

We have used this equation to fit the measured *ΔE(d)* surface peak shift data of Fig. 3 **a** of the main text, and obtained the solid line in Fig. 3 **c**, and therefore the *d*-dependent strain, as expressed in the right vertical axis of the same figure. Note, however, that the solid line fits the set of data at low *1/d* values. In fact, at large *1/d* values the *S* emission from *n*=7 atoms that is expected at lower binding energy nominally becomes a relevant contribution to the *T* peak. Therefore, experimental *ΔE(d)* values cannot be properly associated to a given strain through the effective coordination model using *n*=9.

Supplementary Note 4: Calculations of the CO adsorption on Pt(111) and vicinal Pt(111) surfaces

Comparative *ab initio* calculations have been performed for CO molecules adsorbed on Pt(111), Pt(557), Pt(335), Pt(223) and Pt(221). The observed trends generally agree with previous theoretical analysis using comparable methodologies. The hierarchy of adsorption energies at the Pt(111) plane goes from hollow, to top, and to bridge, being at variance with the experimental observation. [11]. Adsorption energies at the steps are substantially larger than on the terrace. On-top adsorption energy increases by ~450 meV both on A and B steps at low coverages. A similar stabilization is found for bridge adsorption on A steps. However, adsorption on bridge sites on B steps is only 200-300~meV more favorable than on the terrace. This is in accordance with the uptake experiments in Supplementary Fig. 6: on A steps adsorption on both on-top and bridge sites is observed even at low coverage, while only on-top sites appear for B steps. At increasing coverages (we have explored 0.5 ML for all the substrates mentioned above) we find evidence of a long-range repulsion among the molecules with a clear reduction of the adsorption energies in all sites. As expected, adsorption at step edges continues to be largely more favorable than on the terrace.

We have also explored the shifts of C 1s core-level as a function of the adsorption site, finding results consistent both with experiment and previous theoretical studies [12-14]. However, to the best of our knowledge, a systematic study of the dependence of the C 1s CLS as a function of the miscut angle that could be compared with our experimental results is still lacking. Unfortunately this is quite involved since, at variance with the results presented previously for the Pt 4f level, in this case a global reference is missing (such as the bulk Pt in the previous case), to safely compare results computed using supercells of different sizes. For this reason, we decided to analyze the effect of the strain of the Pt surface layer on the C 1s level of adsorbed CO molecules following an approach similar to the *terrace compression* model presented in Supplementary Note 2. However, in this case the adsorption distance of the CO molecule is optimized as function of the applied strain. From these calculations we estimated the shift of the C 1s

level as a function of the strain of the underlying Pt surface layer. Unfortunately, using these data we arrive to the conclusion that the C 1s level for the CO molecule adsorbed on *T* sites should move to lower energies (higher binding energies) as we increase the miscut angle. This is in contradiction with the experimental result, suggesting that either the calculation method or the central assumption that the strain of the terrace is the main factor determining the shift of the C 1s binding energy are faulty.

However, we should be a bit more careful in the previous analysis. We have assumed that the average strain in the terraces is not affected by the adsorption of ~0.5 ML (estimated saturation coverage) of CO. This is against well-known experimental evidence [15] and theoretical results [16, 17], which indicate that the tensile stress of Pt(111) is largely relieved by adsorption (and, which is somewhat more surprising, quite independently on the donor or acceptor character of the adsorbed species). Under these conditions, we can expect that the compression of the terrace will be smaller once it is covered with CO. Indeed, this is confirmed by our calculations, where we observe a reduction of both the in-plane and out-of-plane strains upon CO adsorption. The CO adsorption also produces a noticeable buckling of the surface. Finally and more importantly, in clear contrast to the case of the clean vicinal surfaces, we find a small increase of the average nearest-neighbor distance for the Pt atoms in the terrace as a function of the vicinal angle.

We can now revisit the issue of the C 1s level shift bearing in mind the opposite behavior of the average Pt-Pt distances as compared to the case of the clean vicinal surfaces. This can help to reconcile the data of our simple *terrace compression* model with the experimental observation. Unfortunately a detailed comparison is involved, since the structural changes are more complex for the CO covered than for the clean surface, and will not be attempted here. However, from the analysis presented above, it is tempting to interpret the shift of C 1s level that we observe experimentally as an indication that the relief of the compressive stress induced by CO adsorption is larger for smaller terraces, i.e., for larger miscut angles.

Supplementary References

[1] Björneholm, O.; Nilsson, A.; Tillborg, H.; Bennich, P.; Sandell, A.; Hernnäs, B.; Puglia, C.; Martensson, N. Overlayer structure from adsorbate and substrate core level binding energy shifts: CO, CCH3 and O on Pt(111). Surface Science **1994**, 315, L983–L989.

[2] Wang, J.; Li, W.; Borg, M.; Gustafson, J.; Mikkelsen, A.; Pedersen, T.; Lundgren, E.; Weissenrieder,J.; Klikovits, J.; Schmid, M. et al. One-Dimensional PtO2 at Pt Steps: Formation and Reaction with CO. Physical Review Letters **2005**, 95, 256102.

[3] Tränkenschuh, B.; Fritsche, N.; Fuhrmann, T.; Papp, C.; Zhu, J. F.; Denecke, R.; Steinrück, H. P. A site-selective in situ study of CO adsorption and desorption on Pt(355). The Journal of Chemical Physics **2006**, 124, 074712.

[4] Tränkenschuh, B.; Papp, C.; Fuhrmann, T.; Denecke, R.; Steinrück, H. P. The dissimilar twins – a comparative, site-selective in situ study of CO adsorption and desorption on Pt(322) and Pt(355). Surface Science **2007**, 601, 1108–1117.

[5] Shimizu, S.; Noritake, H.; Koitaya, T.; Mukai, K.; Yoshimoto, S.; Yoshinobu, J. Site specific chemical states of adsorbed CO on Pt(997): A high resolution XPS study. Surface Science **2013**, 608, 220–225.

[6] Gustafson, J.; Borg, M.; Mikkelsen, A.; Gorovikov, S.; Lundgren, E.; Andersen, J. N. Identification of Step Atoms by High Resolution Core Level Spectroscopy. Physical Review Letters **2003**, 91, 056102.

[7] Bianchettin, L.; Baraldi, A.; de Gironcoli, S.; Vesselli, E.; Lizzit, S.; Petaccia, L.; Comelli, G.; Rosei, R. Core level shifts of undercoordinated Pt atoms. Journal of Chemical Physics **2008**, 128, 114706.

[8] Caprile, L.; Cossaro, A.; Falletta, E.; Della Pina, C.; Cavalleri, O.; Rolandi, R.; Terreni, S.; Ferrando, R.; Rossi, M.; Floreano, L. et al. Interaction of l-cysteine with naked gold nanoparticles supported on HOPG: a high resolution XPS investigation. Nanoscale **2012**, 4, 7727–7734.

[9] Köhler, L.; Kresse, G. Density functional study of CO on Rh(111). Phys. Rev. B **2004**, 70, 165405.

[10] Foiles, S. M.; Baskes, M. I.; Daw, M. S. Embedded-atom-method functions for the fcc metals Cu, Ag, Au, Ni, Pd, Pt, and their alloys. Phys. Rev. B **1986**, 33, 7983–7991.

[11] Feibelman, P. J.; Hammer, B.; Nørskov, J. K.; Wagner, F.; Scheffler, M.; Stumpf, R.; Watwe, R.; Dumesic, J. The CO/Pt(111) Puzzle. The Journal of Physical Chemistry B **2001**, 105, 4018–4025.

[12] Zeng, Z.; Ma, X.; Ding, W.; Li, W. First-principles calculation of core-level binding energy shift in surface chemical processes. Science China Chemistry **2010**, 53, 402–410.

[13] Trinh, Q. T.; Tan, K. F.; Borgna, A.; Saeys, M. Evaluating the Structure of Catalysts Using Core-Level Binding Energies Calculated from First-Principles. Journal of Physical Chemistry C **2012**, 117, 1684–1691.

[14] Toyoshima, R.; Yoshida, M.; Monya, Y.; Kazuma, S.; Amemiya, K.; Mase, K.; Mun, B. S.; Kondoh, H. A high-pressure-induced dense CO overlayer on a Pt(111) surface: a chemical analysis using in situ near ambient pressure XPS. Phys. Chem. Chem. Phys. **2014**, 16, 23564.

[15] Ibach, H. The role of surface stress in reconstruction, epitaxial growth and stabilization of mesoscopic structures. Surface Science Reports **1997**, 29, 195 – 263.

[16] Feibelman, P. J. First-principles calculations of stress induced by gas adsorption on Pt(111). Phys. Rev. B **1997**, 56, 2175.

[17] Tian, Z.; Sander, D.; Negulyaev, N. N.; Stepanyuk, V. S.; Kirschner, J. H- and O-induced compressive surface stress on Pt(111): Experiments and density functional theory calculations. Phys. Rev. B **2010**, 113407.
